# Understanding Collective Human Mobility Spatiotemporal Patterns on Weekdays from Taxi Origin-Destination Point Data

**DOI:** 10.3390/s19122812

**Published:** 2019-06-24

**Authors:** Jing Yang, Yizhong Sun, Bowen Shang, Lei Wang, Jie Zhu

**Affiliations:** 1 Key Laboratory of Virtual Geographic Environment (Nanjing Normal University), Ministry of Education, Nanjing 210023, China; YangJing_NNU@163.com (J.Y.); 161302087@stu.njnu.edu.cn (B.S.); 181302096@stu.njnu.edu.cn (L.W.); 2 School of Geography, Nanjing Normal University, Nanjing 210023, China; 3 College of Civil Engineering, Nanjing Forestry University, Nanjing 210037, China; Chu_Je@163.com; 4 Jiangsu Center for Collaborative Innovation in Geographical Information Resource Development and Application, Nanjing 210023, China

**Keywords:** taxi OD points, mobility patterns, time series similarity measurement, driving mechanism

## Abstract

With the availability of large geospatial datasets, the study of collective human mobility spatiotemporal patterns provides a new way to explore urban spatial environments from the perspective of residents. In this paper, we constructed a classification model for mobility patterns that is suitable for taxi OD (Origin-Destination) point data, and it is comprised of three parts. First, a new aggregate unit, which uses a road intersection as the constraint condition, is designed for the analysis of the taxi OD point data. Second, the time series similarity measurement is improved by adding a normalization procedure and time windows to address the particular characteristics of the taxi time series data. Finally, the DBSCAN algorithm is used to classify the time series into different mobility patterns based on a proximity index that is calculated using the improved similarity measurement. In addition, we used the random forest algorithm to establish a correlation model between the mobility patterns and the regional functional characteristics. Based on the taxi OD point data from Nanjing, we delimited seven mobility patterns and illustrated that the regional functions have obvious driving effects on these mobility patterns. These findings are applicable to urban planning, traffic management and planning, and land use analyses in the future.

## 1. Introduction

Urban applications for city planning represent one of the most significant areas in ubiquitous computing, which has great application prospects in urban space research [1,2,3]. In the past, because of the limitations of data availability, analytic tools, and computational capabilities [4], urban computing has been focused more on the analysis of urban physical space [5], functional space [6], and urban form [7]. With the availability of travel data, the focus has recently shifted to the dynamics of social networks [8,9], the evolution of social groups [10,11], and the behavior space of residents [12,13]. As the basis of behavior space analysis, the collective human mobility spatiotemporal patterns are a collection of the time and spatial distribution characteristics of travel behavior, which reflects the spatiotemporal dynamic law of urban travel behavior and helps to reveal the relationship between humans and land in urban environments.

The research on urban travel can be divided into the following three aspects: First, the travel is used to explore land use spatial interaction, which makes the urban land no longer isolated, but a dynamic interaction. By analyzing the citizens’ collective dynamics, Peng et al. [14] pointed out that the characteristics of traveling strongly influence urban formation, evolution, and future planning. Based on the trajectory data and POI data, Yuan et al. [15] and Fu et al. [16] identified different urban functional areas by analyzing the characteristics of travel time series. For better understanding the interactions of urban space, Zhong et al. [17] and Liu et al. [18] identified travel patterns from geo-tagged data to research their relationships with the spatial structure (city hubs, centers, and borders) and the urban built environment. Second, urban travel is applied to traffic analysis, management, and planning. Foell et al. [19] indicated that a main prerequisite for the development of personalized recommender systems is accurate knowledge of travel patterns, and they demonstrated that the prediction accuracy of the mobility pattern can be improved by combing personal and population-wide mobility patterns. Giannotti [20] unveiled the high-level collective mobility knowledge from thousands of private car data to support the decisions of mobility and transportation managers. Peng et al. [14] put forward that the knowledge of the mobility pattern is essential in traffic modeling for simulation, forecast, and control. Third, some scholars have revealed the scaling characteristics of mobility. Brockmann et al. [21] found that the distribution of travelling distances decays as a power law, but Liang et al. [22] discovered that the taxis’ travelling displacements in urban areas tend to follow an exponential distribution instead of a power-law. Liang et al. [23] explained the exponential law of intra-urban mobility as a result of the exponential decrease in average population density in urban areas. Although many scholars have devoted themselves to the above aspects, there are still some deficiencies. Most of the existing studies ignored the differences of mobility patterns and regarded their characteristics and effects on land use and transportation as homogeneous, which is not consistent with reality. As suggested by Yuan et al. [15] and Kang et al. [24], the quality of different venues is different, and their functions vary with the time of day, which are caused by the spatiotemporal differences of travel patterns. For traffic analysis based on travel behavior, the majority of authors did not consider the important parameters: the reasons for moving and traffic demands, which are crucial in each mobility choice and can be obtained by analyzing different travel patterns [25,26]. In terms of movement analysis, Liang et al. [23] pointed out that there is no strong evidence that the collective motions have similar characteristics. Previous studies have shown that we can obtain the geographic spatiotemporal information of blocks, the travelling purposes, the traffic demands and different travelling characteristics by interpreting different types of travel pattern [21,27,28,29]. Therefore, the classification of travel patterns is the basis to further explore cities and transportation. Nevertheless, most of the existing studies on the classification of collective human mobility patterns provide only qualitative descriptions, and quantitative classification results are relatively scarce. Peng et al. [14] found only three kinds of travel patterns on weekdays: commuting between home and workplace, traveling from workplace to workplace, and other patterns such as that for leisure activities. Qi et al. [30] also recognized only three travel patterns and the corresponding regional functions by using a very simple classification method. However, Yuan et al. [15] put forward a functional region corresponding to a mobility pattern, and the regional functions were diverse. Therefore, the classification of mobility patterns still has shortcomings that need to be further studied to understand the causal mechanism of different patterns for the prediction of travel demand, personalized traffic recommendation systems, location choosing, and the planning and management of urban facilities and services [31,32,33].

As one kind of big geospatial data collected through positioning sensors, taxi trajectory data that record the accurate positions and time stamps of the continuous movements of specific people have been widely used to explore urban development [22,34,35]. Due to the large amount of tracking data, a comprehensive analysis is needed to extract useful information. For a mobility pattern, the Origin and Destination (OD) point information of the taxi that records the location and time of the departures and arrivals of taxi passengers is fully adequate to depict the city structure from a land use perspective [36,37,38], while a route that is usually useful for a traffic analysis is not crucial here [39,40,41]. For instance, Yuan et al. [15] proposed a framework that Discovers Regions of different Functions (DRoF) in a city using both taxi OD data and Points Of Interest (POIs) to understand the urban spatial context. Yue et al. [42] and Wang et al. [43] used the taxi OD matrix to discover attractive areas that people often visit and revealed people’s travel demand in a deeper sense to serve taxi transport management and control. Veloso et al. [44] explored the possibility of predicting the next pick-up area type by studying the relationships between pick-up and drop-off locations and characterizing the scenario between taxi services, which is helpful to realize a personalized traffic recommendation system and traffic forecast. In this paper, we expect to classify more mobility patterns from taxi OD point data to support the in-depth study of urban planning and traffic analysis. In the study of human mobility patterns, it is very important to determine the aggregate unit with the OD point data as the research object. Regular grids and traffic zones [1,45] are the most common aggregate units, but they both have limitations in considering the spatiotemporal distribution characteristics of OD points and weaken the spatial characteristics of travel patterns by dividing the OD points of the same travel into different units. First, due to the complexity of urban morphology, it is difficult to determine the division parameters for a regular grid, which often restricts its application [46]. When a road is divided by a grid, the OD points on the same road will be divided into different research units, resulting in data loss. Hence, the regular grid is only suitable for cities with typical latitude and longitude networks, such as the Beijing downtown area and Manhattan. Second, because the taxi OD points are distributed along both sides of the road and have GPS positioning errors, it is impossible to determine exactly on which side of the road the OD points occur. In addition, the network space of urban roads is unevenly distributed, and the traffic zones outside of cities are obviously larger than the traffic zones in cities. This phenomenon leads to difficulties in data normalization and comparison of characteristics, as well as blurs the travel features. Generally, it is also not ideal to make traffic zones aggregate units. Therefore, this paper proposes establishing an aggregate unit for the taxi OD points with road intersections as the constraint condition through a statistical analysis.

The measurement of a time series of aggregate units is a key technique for pattern classification. The Euclidean distance [47], Dynamic Time Warping (DTW) [48], and Fréchet distance [49] metrics are the three conventional and widely-used proximity measures for time series, but those measures ignore the interdependent relationships among the measurements that characterize the time series behavior; the proximity metric is only based on the closeness of the values [50]. To alleviate this major limitation, Chouakria and Nagabhushan [50] proposed a new dissimilarity index based on an automatic adaptive tuning function to include both the behavior and value proximity measures. However, Chouakria and Nagabhushan’s method is not suitable for identifying the mobility patterns in taxi time series data because of the following two special cases that cannot be solved by their method. First, the travel frequency of aggregate units with the same mobility pattern varies greatly. Second, the statistical value of the time node represents the travel amount at the time of the measurement of the taxi time series. The excessive stretching or displacement of the time axis may lead to semantic errors in the matching result, so the matching range of the time node needs to be constrained.

In this study, we designed a new aggregate unit and improved Chouakria and Nagabhushan’s method according to the particular characteristics of taxi travel and used the DBSCAN clustering method to construct a model that is suitable for classifying the mobility patterns obtained from the taxi OD point data. Finally, we identified seven mobility patterns and found that the regional functions have an obvious driving mechanism for those patterns. The rest of the paper is organized as follows: In Section 2, we describe the study area, the measurement method for the time series, and the classification method for the patterns based on the proximity index. In Section 3, we present the case study experiment conducted in Nanjing. Section 4 discusses the driving mechanism of the patterns. Section 5 presents our conclusions on the experimental results and proposes future work.

## 2. Materials and Methods

**Figure 1:**
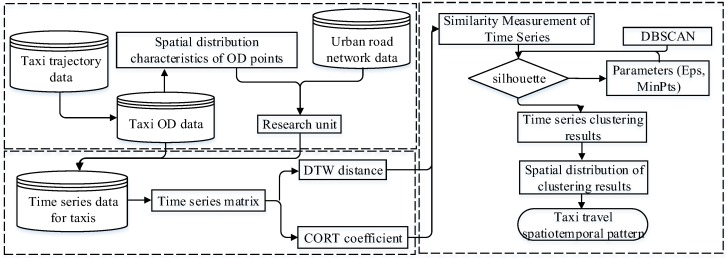
Overview of the procedure for collective human mobility spatiotemporal pattern recognition.

In this paper, we present a model for analyzing the collective human mobility spatiotemporal patterns from taxi OD point data. The proposed model involves the development of an aggregate unit, similarity measurement, and pattern classification and provides a basis for further understanding the driving mechanism of different travel patterns. The general structure of the collective human mobility pattern classification model is illustrated in Figure 1.

### 2.1. Study Area and Data Sources

Nanjing, as the capital of Jiangsu Province in China, is located in the lower reaches of the Yangtze River. Its geographical coordinate range is 31°14′–32°37′ latitude and 118°22′–119°14′ longitude. The total area is 6587.02 km^2^, of which the built-up area is 1399 km^2^, and its urbanization rate has reached 82.50%. From a high altitude view, Nanjing City spans the banks of the Yangtze River and is divided into the Jiangbei area and Jiangnan area, which consist of 11 municipal districts. This paper selected the central urban areas, the Xuanwu, Qinhuai, Gulou, Jianye, Yuhuatai, Jiangning, Qixia, Baixia, Xiaguan, and Pukou districts, as the study areas.

**Figure 2:**
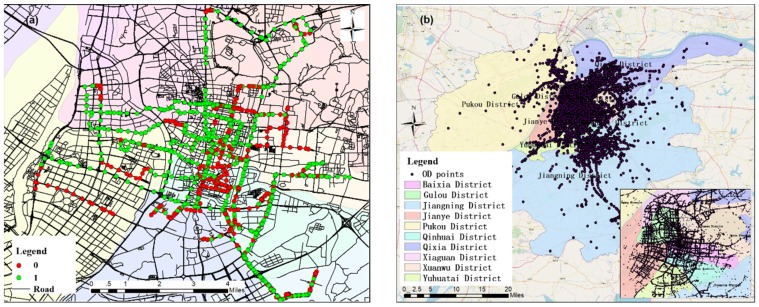
Illustration of the study area (Nanjing, China): (**a**) the spatial distribution of the taxi one-day tracking points, (**b**) the spatial distribution of the taxi OD points and the 10 districts in Nanjing. OD, origin-destination.

The data came from the Nanjing trajectory data of approximately 7700 taxis operating on weekdays from 1–15 September 2010, and the Nanjing road network data were from OSM. The trajectory data for a taxi on a single day include the vehicle status (the “empty state” is 0, and “passenger status” is 1), GPS time, and the latitude and longitude coordinate information. If the taxi tracking point was projected onto a timeline, the passenger status of the taxi could be represented by a series of consecutive 0s and 1s (Figure 2a). When a taxi status changed from no-load to carrying a passenger, this means that a passenger was boarding the taxi. This time was the passenger’s departure time, and the coordinate point was an origin point (O). Otherwise, when the taxi status changed from carrying a passenger to no-load, this means that a passenger exited the taxi. This time was the passenger’s arrival time, and the coordinate point was the passenger’s destination point (D). From the data of 77,000 taxi trajectories, we extracted a total of 2.094 million OD points (Figure 2b).

### 2.2. Travel Time Series Similarity Measurement

By counting the hourly frequency of taxi OD points in a unit, the time series of departures and arrivals over 24 h can be obtained. The similarity measure is a method for calculating the similarity of these travel time series and is the basic algorithm for a travel time series classification analysis. Based on the morphological characteristics of the taxi time series curve, this paper calculated the similarity of the time series of an aggregate unit by using the time series similarity measure function that combines the distance measurement and the similarity measurement of the growth trend.

#### 2.2.1. Determination of an Aggregate Unit

In a study of human mobility patterns, the OD points need to be collected according to the aggregate units, and then, the aggregate model is established based on the aggregate data of the units. Because the traditional aggregate unit, a regular grid and the traffic zone, cannot take into account the spatial and temporal distribution characteristics of the taxi OD data, this paper attempted to introduce the road intersection as a constraint and construct a method suitable for processing the taxi data.

**Figure 3:**
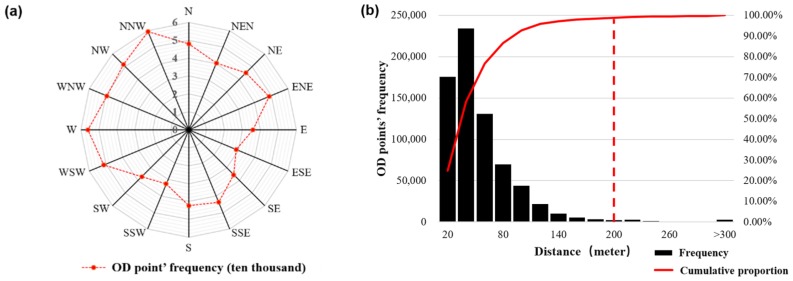

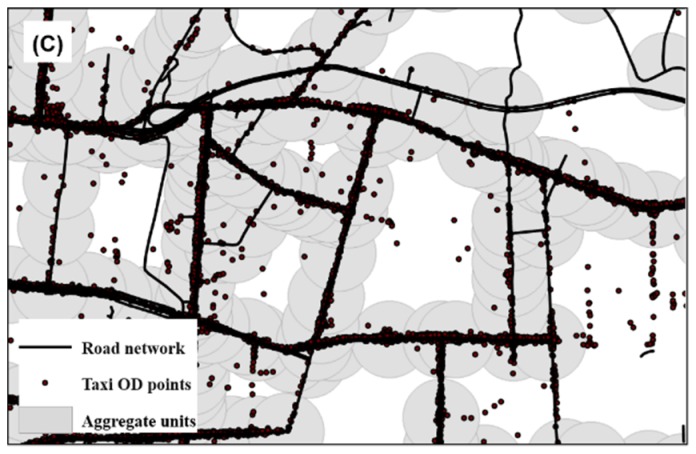
Illustration of the aggregate unit: (**a**) chart of the OD frequency in each direction of the road intersection, (**b**) chart of the distances from the OD points to the nearest road intersection, and (**c**) diagram of the aggregate unit.

A total of 7809 road intersections were extracted from the road network data in the study area. An analysis of the attribute information, such as the distance and direction of the OD points to the nearest road intersection, is shown in Figure 3a. The direction from the OD point to the nearest road intersection was 0° in the northern direction, and the 16-azimuth direction was divided into intervals of 22.5°. The red line records the frequency of the OD points in each direction of the road intersection, and its shape is square-like. The distribution of the OD points in the four vertex directions was the highest, which was roughly consistent with the road direction in the study area. In other directions, the OD points in the opposite direction were similar in frequency, indicating that the taxi OD points were evenly distributed around the road intersections. Figure 3b shows that the highest frequency distance between an OD point and the nearest road intersection was in the range of 20–40 m. As the distance increased, the frequency of the statistically-obtained OD points decreased rapidly. According to the cumulative proportion, the proportion of OD points within 200 m of a road intersection was 98.8%. The analysis of the OD points beyond the 200-m range of a road intersection showed that most of them were in uncovered road network areas such as rivers and mountains, which were probably errors caused by GPS signal drift.

Therefore, as shown in Figure 3c, this paper selected the buffer of a road intersection with a bandwidth of 200 m as the research unit, used the OD frequency of taxis as the statistical indicator to collect the processing results, and constructed a time series dataset using one-hour intervals.

#### 2.2.2. Distance Function Based on Dynamic Time Warping

**Figure 4:**
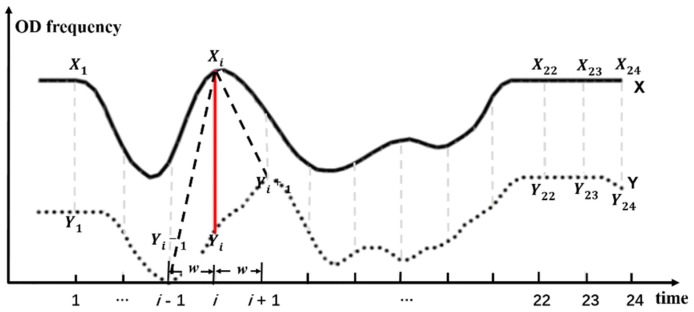
Diagram of the DTW distance function considering the time window (DTW, dynamic time warping).

The DTW distance is used to find the best alignment and matching distance between time series as a measure of time series distance. The smaller the value is, the higher the similarity is. As shown in Figure 4, X and Y were two taxi travel time series (O), each generating 24 time node pairs, expressed as $\left[\left(X_{1},\;Y_{1}\right),\left(X_{2},\;Y_{2}\right),\;\ldots ,\left(X_{24},\;Y_{24}\right)\right]$.

Then, the distance between the pairs of time nodes was calculated, and the distance matrix was constructed. In $D\left(Fre_{X_{i}},Fre_{Y_{j}}\right)=\left|Fre_{X_{i}}-Fre_{Y_{j}}\right|$, $Fre_{X_{i}}$ and $Fre_{Y_{j}}$ represent the travel frequency corresponding to the respective time nodes. $D\left(Fre_{X_{i}},Fre_{Y_{j}}\right)$ is the absolute difference between the travel frequencies of time nodes $X_{j}$ and $Y_{j}$, indicating the distance between different time node pairs.

$$D=\left[\begin{array}{cccc} Fre_{X_{1}},Fre_{Y_{1}} & Fre_{X_{2}},Fre_{Y_{1}} & \ldots & Fre_{X_{24}},Fre_{Y_{1}}\\ Fre_{X_{1}},Fre_{Y_{2}} & Fre_{X_{2}},Fre_{Y_{2}} & \ldots & Fre_{X_{24}},Fre_{Y_{2}}\\ \ldots . & \ldots . & Fre_{X_{i}},Fre_{Y_{j}} & \ldots \\ Fre_{X_{1}},Fre_{Y_{24}} & Fre_{X_{2}},Fre_{Y_{24}} & \ldots & Fre_{X_{24}},Fre_{Y_{24}} \end{array}\right]$$

The DTW distance supports the time series matching by time axis scaling and displacement, but in the condition of the taxi time series, since the statistical value corresponding to the time node expressed the traveling frequency of the time node, the excessive stretching or displacement of the time axis may lead to semantic errors in the matching results, so it is necessary to limit the scope of the time axis expansion. This situation can be avoided by adding a time window and effectively reducing the time complexity of the algorithm. In this paper, the time interval of the taxi time series dataset was 1 h. To preserve the morphological characteristics of the time series during the matching process, the value of the DTW time window, w, was 1 (Figure 4). Based on the recursive relationship, using Equation (1), the minimum value of the cumulative distances in the distance matrix was the DTW distance.

(1) $$D_{dtw}\left(Fre_{X_{i}},Fre_{Y_{j}}\right)=D\left(Fre_{X_{i}},Fre_{Y_{j}}\right)+min\{\begin{array}{c} D_{dtw}(Fre_{X_{i-1}},Fre_{Y_{j-1}})\;\\ D_{dtw}\left(Fre_{X_{i-1}},Fre_{Y_{j}}\right)\;\left|i-j\right|\leq 1\\ D_{dtw}\left(Fre_{X_{i}},Fre_{Y_{j-1}}\right)\; \end{array}.$$

where $D_{dtw}\left(Fre_{X_{i}},Fre_{Y_{j}}\right)$ represents the DTW distance (similarity) between the band of time series X before time node $X_{i}$ and the band of time series Y before time node $Y_{j}$. $D_{dtw}\left(Fre_{X_{24}},Fre_{Y_{24}}\right)$ indicates the similarity between the overall waveforms of time series X and Y.

#### 2.2.3. Adaptive Dissimilarity Index

The DTW distance calculates the similarity of two time series by the degree of approximation of the values. However, there are correlations among the values of each time node in the time series. The direction and rate of growth of the values also express the similarity of the time series [51]. Chouakria and Nagabhushan [50] introduced a method for measuring the time series growth model, the purpose of which was to calculate the correlation coefficient of the growth trend of the time series to correct the time series distance obtained by the conventional metric method, which is also called the adaptive dissimilarity index, as shown in Equation (2).

(2) $$CORT\left(Fre_{X_{24}},Fre_{Y_{24}}\right)=\frac{\sum_{i=1}^{23}\left(Fre_{X_{i+1}}-Fre_{X_{i}}\right)\left(Fre_{Y_{i+1}}-Fre_{Y_{i}}\right)}{\sqrt{\sum_{i=1}^{23}{\left(Fre_{X_{i+1}}-Fre_{X_{i}}\right)}^{2}}\sqrt{\sum_{i=1}^{23}{\left(Fre_{Y_{i+1}}-Fre_{Y_{i}}\right)}^{2}}}.$$

The range of $CORT\left(Fre_{X_{24}},Fre_{Y_{24}}\right)$ is [−1, 1]. $CORT\left(Fre_{X_{24}},Fre_{Y_{24}}\right)=1$ indicates that the two time series, X and Y, have the same growth direction and growth rate at each time node. $CORT\left(Fre_{X_{24}},Fre_{Y_{24}}\right)=-1$ indicates that the two time series’ growth rates are the same, but are in opposite directions. $CORT\left(Fre_{X_{24}},Fre_{Y_{24}}\right)=0$ indicates that the growth characteristics of time series X and Y are randomly independent. The larger the value of $CORT\left(Fre_{X_{24}},Fre_{Y_{24}}\right)\;\mathrm{is}$, the more similar the two time series are. In the DTW distance function, the more similar the two time series are, the smaller the DTW distance is. Therefore, the exponential adjustment function needs to be used to adjust the adaptive dissimilarity index [52], as shown in the formula:

(3) $$F\left(CORT\left(Fre_{X_{24}},Fre_{Y_{24}}\right)\right)=\frac{2}{1+exp\left(2CORT\left(Fre_{X_{24}},Fre_{Y_{24}}\right)\right)}$$

After the adjustment, the more similar the two time series are, the smaller the adjustment index $F\left(CORT\left(Fre_{X_{24}},Fre_{Y_{24}}\right)\right)$ of the adaptive dissimilarity index is, which is consistent with the change trend of the DTW distance.

#### 2.2.4. Construction of the Similarity Measurement Function

**Figure 5:**
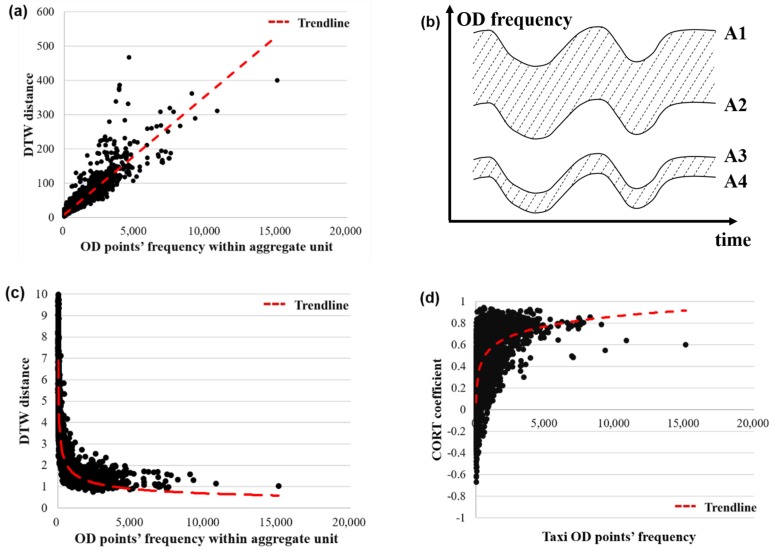
Time series similarity measurement: (**a**) the unnormalized correlation between the DTW distance and the OD frequency, (**b**) the differences in the absolute distances within the same series, (**c**) the normalized correlation between the DTW distance and the OD frequency, and (**d**) the correlation between the CORT coefficient and the OD frequency.

In general, when the amount of travel within a research unit is large, the randomness of the overall travel behavior within the unit is small, and the daily mobility pattern becomes more stable. In the time series, when the amount of travel within a research unit is high, the average DTW distances among the multiple working day time series in the region is small, that is the more similar the time series are. Equation (1) is used to calculate the average DTW distances among the time series of multiple working days in each research unit. As shown in Figure 5a, the frequency of the OD points in the research unit was linearly correlated with the average DTW distance, which is inconsistent with general understanding. This is because the distance between the pairs of the time nodes in the DTW distance formula was calculated by the Euclidean distance. The OD frequencies in the time series dataset may differ by more than several orders of magnitude. As the OD frequency increased, the absolute distance of the two time series increased. As shown in Figure 5b, even though the time series A1, A2, A3, and A4 were exactly the same, the absolute distance between time series A1 and A2 was significantly larger than the absolute distance between time series A3 and A4.

To eliminate the influence of the absolute value of the sample, the original data must be normalized by Equations (4)–(6), where $Fre{}^{\prime }{}_{X_{i}}$ represents the corresponding travel frequency of time node $X_{i}$ after normalization. Using Equation (1), the DTW distance of the normalized taxi time series dataset was recalculated. As shown in Figure 5c, as the OD frequency increased, the research unit’s own DTW distance tended to become stable. The time series distance measurement results were more realistic.

(4) $$\bar{X}=\frac{\sum_{1}^{24}Fre_{X_{i}}}{24}$$

(5) $$S_{X}=\sqrt{\frac{\sum_{i=1}^{24}{\left(Fre_{X_{i}}-\bar{X}\right)}^{2}}{23}}$$

(6) $$Fre{}^{\prime }{}_{X_{i}}=\frac{\left(Fre_{X_{i}}-\bar{\bar{X}}\right)}{S_{X}}$$

Similarly, the adaptive dissimilarity index was verified. Since the adaptive dissimilarity index was calculated by the ratio of the variance sum to the covariance sum, it was insensitive to the absolute value difference of the input time series (Figure 5d), so no normalization was required. With the increase in the OD frequency in the research unit, the CORT coefficient tended to be 1, which indicates that the taxi travel time series tended to be stable on multiple working days in the region, which is consistent with the inference.

In summary, the time series similarity measure function used in this paper can be written as Equation (7), where D(X, Y) is the index of the final evaluation of the sequence similarity, and the smaller the value is, the more similar the time series are. $CORT\left(Fre_{X_{24}},Fre_{Y_{24}}\right)$ is the time series adaptive dissimilarity index, and F(x) is the adjustment index of the CORT coefficient. $D_{dtw}\left(Fre{}^{\prime }{}_{X_{24}},Fre{}^{\prime }{}_{Y_{24}}\right)$ is a DTW distance function with a time window and normalization constraints.

(7) $$D\left(X,Y\right)=F\left[CORT\left(Fre_{X_{24}},Fre_{Y_{24}}\right)\right]\times D_{dtw}\left(Fre{}^{\prime }{}_{X_{24}},Fre{}^{\prime }{}_{Y_{24}}\right)$$

### 2.3. Collective Human Mobility Spatial-Temporal Pattern Recognition

#### 2.3.1. Clustering Method for the Travel Time Series

The DBSCAN (Density-Based Spatial Clustering of Applications with Noise) algorithm is a clustering method that can effectively eliminate dataset noise. The method is based on a set of neighbors determined by a minimum number (MinPts) of samples within the search radius (Eps), and the search radius is configured to describe how closely the sample set and these two parameters together define the minimum density threshold of the neighborhood. The portion of the sample density greater than the neighborhood density threshold will be identified and clustered, and the portion less than the neighborhood density threshold will be considered noise. Assuming that there are n time series, using the above similarity measure function, each time series has n−1 similarity indexes compared with other time series. Taking any one time series as the kernel sequence, when the similarity index value of at least MinPts time series in the n−1 similarity index is less than Eps, the time series in the search radius will be grouped into a cluster, which corresponds to a mobility pattern.

The silhouette technique is a method to evaluate the quality of the clustering results based on the degree of compactness within the clusters and the degree of separation between the clusters [53]. For the $i^{th}$ time series in the cluster, calculate the average similarity index of the other time series within the cluster, denoted as *a*(*i*), and calculate the minimum similarity index of the time series with the other clusters as *b*(*i*). For time series *i*, the contour coefficient can be expressed by Equation (8):

(8) $$S\left(i\right)=\frac{b\left(i\right)-a\left(i\right)}{max\left\{a\left(i\right),b\left(i\right)\right\}}$$

The contour coefficient, *S*(*i*), has a value range of [−1, 1], and when *S*(*i*) approaches 1, it indicates that time series *i* is clustered into a suitable cluster. The contour coefficients of the clusters are expressed by the mean values of the contour coefficients of each time series within the cluster. Therefore, when the contour coefficient of the clustering result is close to 1, the clustering effect is better [15].

In this experiment, the iterative method was used to test the contour coefficients of the clustering results under different combinations of the Eps and MinPts parameters, and the minimum density threshold of the neighborhood was obtained to identify the collective human mobility spatiotemporal pattern from the time series.

#### 2.3.2. Comparing the Results with the K-Means Method 

**Figure 6:**
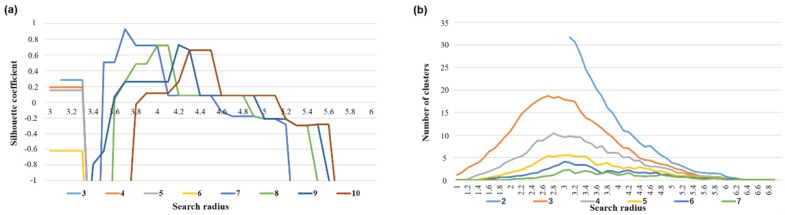
Determining the threshold value: (**a**) the correlation between the Eps value and the contour coefficient with different MinPts values and (**b**) the correlation between the Eps value and the number of clusters with different MinPts values.

To verify the method described in this section, the top 200 research units with the highest frequency of travel on working days were selected as the sample dataset and were clustered based on the similarity index among the time series of the research units. First, the DBSCAN clustering threshold was determined. As shown in Figure 6, the abscissa is the Eps value, and each color curve represents a clustering result corresponding to different MinPts values. When the Eps value was 3.7 and the MinPts was 7, the clustering result contour coefficient of the sample dataset reached a maximum value of 0.91, and the optimal threshold value for the time series dataset was [Eps = 3.7, MinPts = 7], while the corresponding number of clusters was 3. The K-means clustering algorithm was used to cluster the same sample dataset by setting 3 clusters.

**Figure 7:**
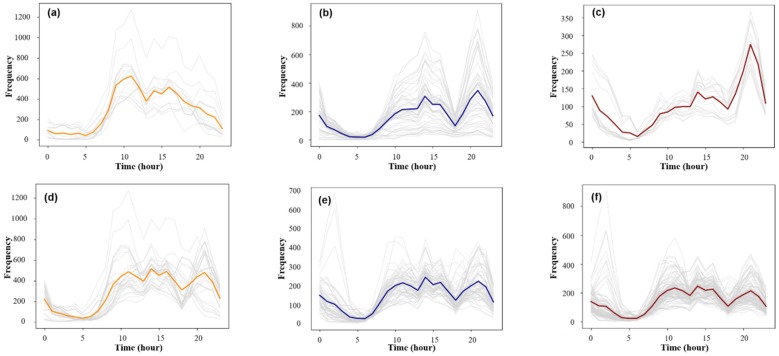
Clustering results of the simulation experiments: (**a**–**c**) the clustering results of the proposed method and (**d**–**f**) the clustering results of the K-means method.

As shown in Figure 7, the clustering results were sorted according to the maximum value of the average frequency of the traffic volume. The results of this method were named Cluster 1, Cluster 2, and Cluster 3, and the results of the comparison experiment were named Contrast 1, Contrast 2, and Contrast 3. Cluster 1 contained 11 samples, Cluster 2 71 samples, and Cluster 3 46 samples, which is a total of 128 samples. The remaining 72 samples were considered as random samples that were treated as noise and excluded. Contrast 1 contained 34 samples, Contrast 2 62 samples, Contrast 3 104 samples; thus, these clustering results contained all 200 samples. Since the K-means algorithm cannot eliminate the interference of noise items, the clustering result is more dependent on the absolute value of the trip frequency for classification. The contour coefficient of the clustering result obtained by the K-means algorithm was 0.32, which indicates that the cohesion and separation were not ideal. Therefore, the clustering results obtained by this method were more suitable for a time series cluster analysis of the taxi travel.

## 3. Results

### 3.1. Screening the Aggregate Units

From the trend line of the average value of Figure 5c, it can be clearly seen that the stability of the taxi mode in the research unit was closely related to the OD frequency. When the frequency of the research unit was less than approximately 500, the average DTW distances among the time series of multiple working days in each unit rose sharply, and the mobility pattern showed instability. To improve the reliability of the subsequent time series clustering results, the original research unit was screened, and only 1902 research units with an average frequency of more than 500 trips per day were analyzed.

### 3.2. Extraction of the Spatiotemporal Travel Patterns

#### 3.2.1. Classification of the Travel Time Series

**Figure 8:**
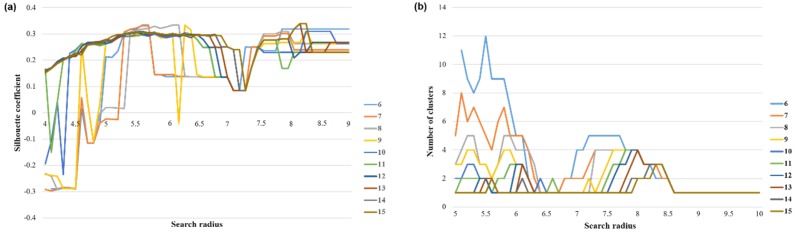
Determination of the clustering threshold value of the departure time series on weekdays: (**a**) the contour coefficients of the clustering results under different threshold combinations and (**b**) the number of clusters under different threshold combinations.

According to the above method, the optimal clustering threshold of the departure and arrival time series in 1902 research units was determined, and the time series was classified. The search radius was from 3–10, the step size 0.1, the minimum sample size from 6–15, and the step size 1; the time series dataset was iteratively clustered 80 times. The clustering contour coefficient and the number of clusters were mapped to the coordinate axes, as shown in Figure 8.

**Figure 9:**
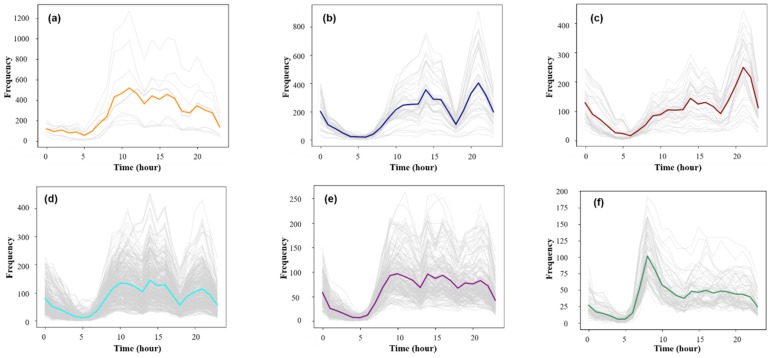

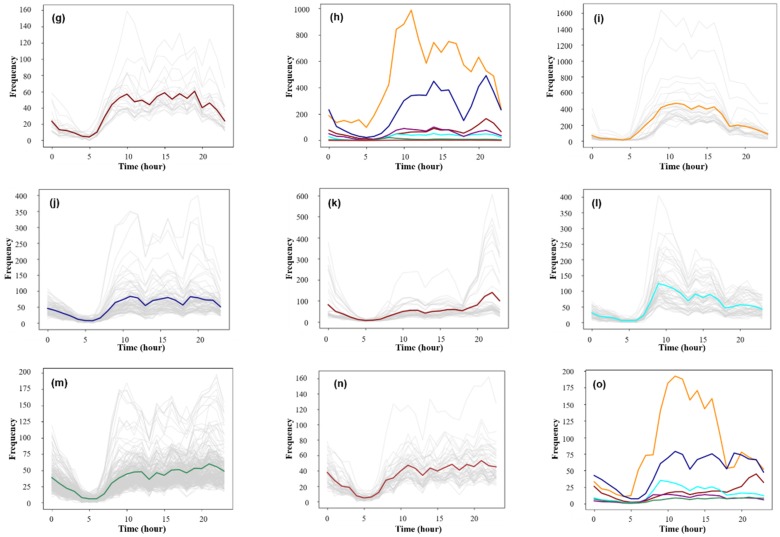
(**a**–**h**) The departure time series of the collective human mobility patterns on weekdays and (**i**–**o**) the arrival time series of the collective human mobility patterns on weekdays.

It can be seen from Figure 8 that when the Eps threshold was 8.2, the contour coefficient was the largest, but only two clusters were obtained. The number of clusters was too small to distinguish the mobility pattern well. Seven clusters were obtained by selecting the threshold value of the second largest contour coefficient (0.33) [Eps = 5.7, MinPts = 7]. The clustering results were sorted from large to small according to the maximum average frequency of the traffic and were named PU1–PU7 (Pick-Up) (Figure 9a–g). Based on the same method, the data of the working day departure time series were clustered. When the threshold value was selected [Eps = 6.1, MinPts = 8], the clustering result contour coefficient was 0.31, corresponding to six clusters. According to the maximum average frequency, the clustering results were named DF1–DF6 (Drop-Off) (Figure 9i–n).

#### 3.2.2. Spatial Distribution of the Travel Patterns

**Figure 10:**
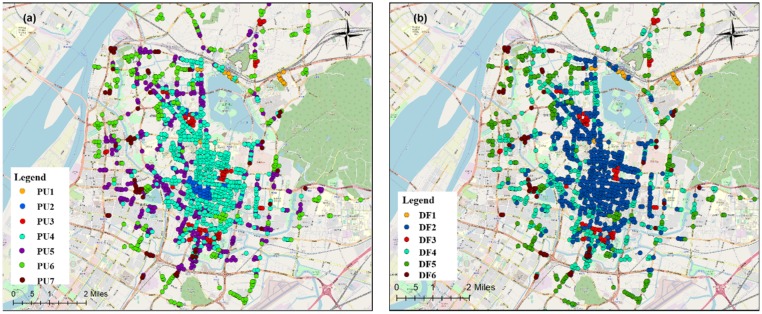
Spatial distribution map of the departure and arrival patterns on weekdays. PU, Pick-Up. DF, Drop-Off.

By using the DBSCAN clustering method to screen out the noise sequences, a total of 1309 effective research units were obtained, and the clustering results of the departures and arrivals on working days were correlated to obtain the spatial distribution map of the human mobility spatiotemporal patterns (Figure 10).

PU1 contained only 21 research units, but it had the highest average daily travel frequency with an average of 400–500 trips per hour, mainly distributed in Nanjing Railway Station, Lukou Airport, two bus stations, and the Gulou subway station. This type of area was at its peak from 09:00–17:00, and there would be a small peak at 20:00. These areas usually have large transportation hubs or important metro stations in the city, and there is a strong and stable demand for taxis. PU2 contained 47 research units, and the frequency of travel was slightly lower than that of PU1, mainly distributed in Xinjiekou and the Hunan Road shopping districts and some subcentral areas. The time series in these areas had two peaks at approximately 14:00 and 21:00 due to work commuting and the travel to return home after entertainment and shopping in the evening. PU3 was highly concentrated in space, distributed near the Confucius Temple, Block 1912, and the Lions Bridge pedestrian street. The peak travel pattern in this area occurred from evening to night, and the number of departure points in the early morning was higher than in other areas. This type of area comprises the major entertainment areas in Nanjing, with demand for evening travel on weekdays. PU4 had the largest number of research units and the largest regional area, including the main commercial areas of Nanjing. There were three peaks in the number of trips in this area. The daytime peak travel coincided with the working hours, which should be the transactional travel between workplaces, while a slight peak in the evening reflected travel for entertainment purposes. The distance from PU5 to the center of the city is farther than that of PU4. The time series were similar, but the fluctuations of PU4 were smaller, which had less travel in the evening and lower overall travel demand. PU6 had 268 areas with a very obvious morning peak travel. The area is far from the center of the city and is one of the newly-built residential quarters around the city. These areas are mainly residential and lack places for employment. Therefore, the travel in such areas is mainly commuting to workplaces. PU7 contained 67 regions, and the spatial distribution was relatively scattered. It usually appeared at the junction of the PU4, PU5, and PU6 regions. The total amount of travel in the mobility pattern was similar to that of PU6. The waveform was stable and should be a mixed residential and work area.

Figure 10b shows the results of clustering the arrival time series data for working days and obtained DF1 with six travel patterns. Combined with Figure 10a, DF1, 3, 5, and 6 were basically associated with PU1, 3, 6, and 7, respectively. DF1 was still the travel pattern with the largest average travel volume, which showed that the transportation hub had a greater attraction for mass travel. The DF2 area is distributed in the center of the city and contains most of the areas of PU2 and PU4 in terms of the departure pattern. The arrival time series of the region fluctuated less with three smaller arrival peaks in the working hours and evenings, which were presumed to be transactional travel between work sites. DF3 and PU3 had similar distribution ranges, and the arrival frequency reached a peak at approximately 22:00, which was basically synchronized with an increase in the departure traffic volume. The DF4 area was basically coincident with the PU5 area and covered a part of the PU4 area, which is far from the city center. Besides, the arrival time series of the region had two distinct peaks. The number of passengers in the DF5 area increased slowly from the afternoon, with two peaks at 17:00 and 21:00, corresponding to the travel demand for the rush hour and the return to residences at night. DF6 had fewer areas, and the daily average trip volume was the lowest among all travel patterns. Its spatial distribution was similar to that of PU7, and it is a transitional zone of various functional areas.

Superimposing the clustering results of the departure and arrival time series for weekdays, PU1, 3, 6, and 7 were combined with DF1, 3, 5, and 6, respectively, in the arrival classification data, and the number of research units in a single combination accounted for more than 80% of the classification results, which can be considered as the same type of travel. The clustering results PU2, 4, and 5 for the departure data and the clustering results DF2 and 4 for the arrival data had various combinations, and the proportion of any single combination did not exceed 70% of the whole. Because of the significant difference in the time series among PU2, 4, and 5 in the departure data clustering results, we used the clustering results of the departure data to determine the spatiotemporal patterns of taxi travel on weekdays and named the travel patterns Mode 1–Mode 7.

## 4. Discussion

The above analysis divided and qualitatively analyzed the taxi travel patterns for working days in Nanjing. To further analyze the relationship between each travel spatiotemporal pattern and the functional features in the corresponding unit, this paper discusses the driving mechanism of the mobility patterns in combination with the 22 POI types in Table 1.

The Random Forest (RF) algorithm [54] is good at identifying the relevant features with only a few major influences on the high-dimensional data and supports the quantitative expression of the characteristics of the influencing factors. In recent years, this algorithm has been widely used in various classification applications and predictions such as feature selection and anomaly detection problems [54,55,56,57,58]. Here, we used the random forest approach to calculate the feature importance and feature contribution metrics to analyze the importance and impact trends of different POIs on the seven mobility patterns. The variable importance metric is a quantitative indicator of the impact of each POI feature on the classification result of the model. The principle is that when the importance of a certain POI is high, the POI is replaced by random noise in the travel pattern classification model. The greater the decrease is in the prediction accuracy, the more the degree of feature importance reflects the importance of the influencing factor to the pattern classification. The feature contribution metric [59] is a quantitative indicator of the contribution of a POI feature to the travel pattern classification results of the random forest algorithm. It reflects the impact of the influencing factor on the classification results of the mobility patterns. In the travel pattern division process, when a certain POI feature contribution score has a positive value, it indicates that the POI feature will increase the probability that the research unit is classified into the travel pattern.

The 22 POIs in Table 1 were used to construct the kernel density surface, and the average kernel density values of the various POIs in each study unit were used as the characteristic values. Second, the eigenvalues of different dimensions were normalized using Equation (9).

(9) $$V{}^{\prime }{}_{X_{i,\;j}}=\frac{V_{X_{i,\;j}}-V_{X_{i,\;min}}}{V_{X_{i,max}}-V_{X_{i,\;min}}}$$

where $V_{X_{i,\;j}}$ represents the mean value of the $X_{i}$ POI kernel density in the study unit j. $V_{X_{i,max}}$ and $V_{X_{i,\;min}}$ represent the maximum and minimum values of the average kernel density of the $X_{i}$ POIs of all the study units, respectively. $V^{\prime }{}_{X_{i,\;j}}$ represents the eigenvalue of the $X_{i}$ POI in study unit j after normalization, and the range of the value was [0, 1].

We selected 70% of the research units as a training set, used the random forest algorithm to calculate the feature importance and feature contribution of the POI eigenvalues in the sample set, and constructed an association model between the two POI metrics and the mobility patterns. The remaining 30% of the research units were used to verify the association model, and the metric-based travel pattern classification result confusion matrix was obtained (Table 2).

Based on the confusion matrix of the classification results, the F1 score [60] of each travel pattern class was calculated, which can be used to determine the classification accuracy of each mobility pattern. As shown in Table 3, except for the lower scores of Modes 4 and 7, the classification accuracies of the other travel patterns were greater than 70%, and the model’s overall accuracy (weighted average) reached 72%. The results showed that the POI features selected in this paper were related to the mobility pattern, reflecting that the regional function had a driving effect on the taxi mobility pattern.

**Figure 11:**
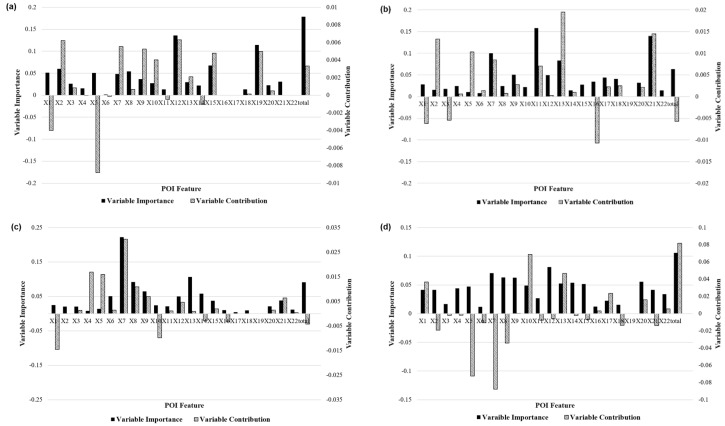

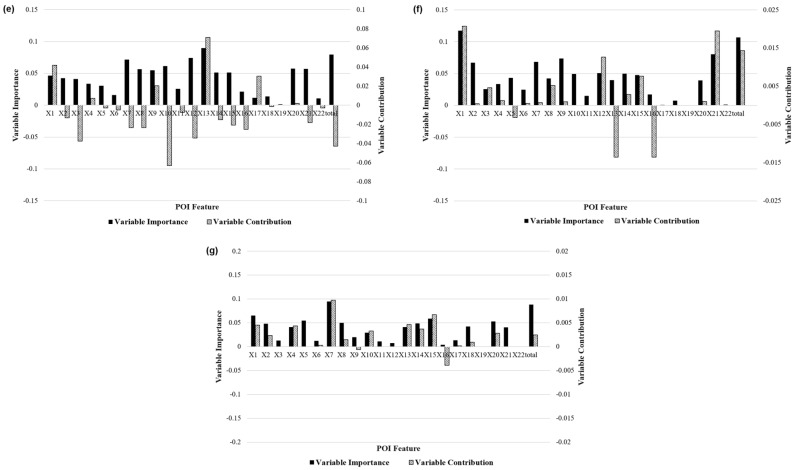
Driving mechanisms of the different travel patterns on weekdays: (**a**–**g**) the feature importance and feature contribution metrics of different POIs of mode1–mode7. POIs, Points of interest.

Figure 11 further analyzes the feature importance and feature contributions of various travel pattern driving mechanisms. Each black bar in the graph indicates the feature importance, and striped bar indicates the feature contribution. In general, the residential (X1) and commercial (X7–17) features obtained higher scores in the characteristic importance and contribution of most taxi patterns, while the transportation facilities (X18–21) had a lower contribution to each pattern, mainly because these type of POIs were more relevant to bus and subway travel and had less impact on taxi travel. The traffic hub feature (X19) accounted for a very low proportion of the overall feature importance analysis of the model, but it had a large positive contribution to the Mode 1 class. Modes 1 and 2 were very similar after deleting such features. In the Mode 3 category, the characteristic importance and contribution of the restaurants, hotels, and entertainment (X7–9) achieved higher scores, and the catering characteristics had an importance of more than 20%. This phenomenon explains why these areas had more night trips. In the Mode 6 category, the feature importance of the residential community (X1) was 12%, which was significantly higher than the characteristic importance of the residences in all other categories, and the characteristic contribution of the service facilities (parking and retail) of the residential communities in the region also achieved a high score, which verifies that the area was mainly residential. Modes 4 and 5 were also highly correlated with commercial buildings and corporate features, indicating that the area was in a mixed working and residential living area. The importance and characteristics of the various features in Mode 7 were similar, and there were no obvious features. This is because the region was in the transitional zone of various functional areas, which is also the reason why the number of ODs in the regional travel pattern was small.

## 5. Conclusions

In this paper, we built buffers based on road intersections as the research units. The taxi trajectory data were collected and processed, and the taxi travel time series dataset was constructed. Then, we constructed a travel time series dataset from the taxi trajectory data by using an aggregate model. Based on the time series characteristics of the spatiotemporal patterns of taxi travel, the taxi mobility patterns were identified and extracted based on the time series clustering method. Using a random forest algorithm to establish a correlation model between the mobility patterns and the POI data, a quantitative analysis of the travel pattern driving mechanism was obtained. The main conclusions of this paper are as follows:

- We used the DBSCAN algorithm, which can effectively eliminate noise, to cluster the taxi travel time series data, and seven departure patterns and six arrival patterns were obtained. Finally, seven human mobility patterns were delimited through spatial matching based on the aggregate units.
- Using the random forest algorithm, this paper established a correlation model between the mobility patterns and POI features. Using the feature importance and feature contribution measures as indicators, it was verified that the different urban regional functions had different driving mechanisms for the various taxi travel patterns.

The taxi OD point data analyzed in this study are only applicable to study the travel associated with the central city. Buses or other modes are used for long-distance travel and most cases of commuting, and these modes are expected to incorporate intelligent public transport data to improve urban travel and extend the recognition range of the spatiotemporal modes. In addition, when using the POI data to analyze the driving mechanism of the travel patterns, this paper did not consider attributes such as the POI level, area, or influence range. In the future, it will be necessary to add the survey data, such as the land use status data, to further improve the accuracy of the travel pattern driving mechanism analysis.

## Figures and Tables

**Table 1:** POI feature set and land use types.

Feature ID	Feature Description	Urban Construction Land Category
X1	Residential area	Residential
X2	Government agency	Administrative office
X3	Cultural facilities	Cultural facility
X4	School/research institute	Educational /Research
X5	Hospital	Medical
X6	Scenic area	Cultural relics and historic sites; religious facilities
X7	Restaurant	Commercial facility
X8	Hostel	Commercial facility
X9	Entertainment venue	Commercial facility
X10	Supermarket	Commercial facility
X11	Department store	Commercial facility
X12	Retail store	Commercial facility
X13	Commercial Building	Commercial facility
X14	Bank	Commercial facility
X15	Company	Commercial facility; Industrial
X16	Cinema	Recreation and wellness facilities
X17	Wellness facility	Recreation and wellness facilities
X18	Subway station	Urban rail transit
X19	Transportation hub	Transportation hub
X20	Bus station	Traffic station site
X21	Parking lot	Traffic station site
X22	Park/garden	Green space

**Table 2:** The confusion matrix of the taxi mobility pattern classification results.

	Simulation Mode	Mode 1	Mode 2	Mode 3	Mode 4	Mode 5	Mode 6	Mode 7
Actual Mode								
Mode 1		7	0	0	1	1	0	0
Mode 2		0	9	0	0	6	0	0
Mode 3		0	0	7	0	5	0	0
Mode 4		0	0	0	22	14	1	1
Mode 5		0	0	0	15	92	4	0
Mode 6		0	0	0	1	2	15	0
Mode 7		0	0	0	3	3	1	2

**Table 3:** Accuracy of the classification model for the weekday travel patterns.

Mode	Precision	Recall	F1-Score	Support
Mode 1	1.00	0.78	0.88	9
Mode 2	1.00	0.60	0.75	15
Mode 3	1.00	0.58	0.74	12
Mode 4	0.52	0.58	0.55	38
Mode 5	0.75	0.83	0.79	111
Mode 6	0.71	0.83	0.77	18
Mode 7	0.67	0.22	0.33	9
Weighted average	0.74	0.73	0.72	212
